# Does the Stereotypicality of Mothers’ Occupation Influence Children’s Communal Occupational Aspirations and Communal Orientation?

**DOI:** 10.3389/fpsyg.2021.730859

**Published:** 2021-12-16

**Authors:** Marie Kvalø, Marte Olsen, Kjærsti Thorsteinsen, Maria I. T. Olsson, Sarah E. Martiny

**Affiliations:** ^1^Department of Psychology, Research Group Social Psychology, UiT The Arctic University of Norway, Tromsø, Norway; ^2^Department of Psychology, Inland School of Business and Social Sciences, Inland Norway University of Applied Sciences, Lillehammer, Norway

**Keywords:** mothers’ occupation, career development, elementary school children, role models, occupational aspirations

## Abstract

Career development is a lifelong process that starts in infancy and is shaped by a number of different factors during childhood, adolescence, and adulthood. Even though career development is shaped through life, relatively little is known about the predictors of occupational aspirations in childhood. Therefore, in the present work we investigate how the stereotypicality of a mother’s occupation (female-dominated/communal vs. non-female-dominated/agentic) influences her young child’s communal occupational aspirations and communal orientation. We conducted two studies with young children. Study 1 included 72 mother–child dyads recruited from childcare centers in Northern Norway (children’s age range: 4½–6 years). Study 2 included 106 mother–child dyads recruited from Norwegian elementary schools (children’s age range: 6 to 13 years). Results from Study 1 showed that the stereotypicality of mothers’ occupation was related to their children’s communal occupational aspirations and children’s communal orientation. In contrast to our predictions and results from Study 1, the stereotypicality of mothers’ occupation was not significantly related to children’s communal occupational aspirations nor their communal orientation in Study 2. In both studies, we found no relationship between mothers’ gender attitudes or share of child care and children’s communal occupational aspirations. The results are discussed in terms of parents’ influence on children’s development of occupational aspirations.

## Introduction

Occupational aspirations develop across all stages of life, from infancy to childhood, adolescence, and adulthood (e.g., Gottfredson, 1981; Barak et al., 1991; Hartung et al., 2005). Though researchers agree that occupational aspirations develop across the lifespan, most research has focused on adolescence and young adulthood, and relatively little is known about career development in both early and middle childhood. As the main attachment figures for young children, parents can be considered one of the main sources of influence in the development of occupational aspirations and communal and agentic orientation in early childhood. Therefore, the present study investigates parental influence on young children’s communal occupational aspirations and their communal orientation. Communality is defined as a stereotypically feminine dimension of traits such as being kind, thoughtful, and sensitive to others’ feelings (Rudman and Glick, 2001) that are particularly important for occupations such as nursing and teaching in early education (Diekman et al., 2010). In the present work, we focus on the mothers’ role in influencing their children. Specifically, we investigate whether the stereotypicality of mothers’ occupations, their share of child care with their partner, and their gender attitudes are associated with their children’s communal occupational aspirations and communal orientation.

In the present work, we focus on aspirations of young children toward communal roles because researchers have argued that more knowledge is needed about which factors promote boys’ and men’s interest in communal roles (Croft et al., 2015; Van Grootel et al., 2018). Increasing the number of men in communal roles has several benefits to society as well as to individuals. At an organizational and societal level, increasing the number of men entering communal work can broaden the diversity in these occupations and meet the labor shortages in care fields (Croft et al., 2015). At an individual level, having a communal orientation has benefits such as increasing individuals’ happiness and satisfaction with life (Fleeson et al., 2002; Sheldon and Cooper, 2008; Bauer and McAdams, 2010; Holt-Lunstad et al., 2010; Le et al., 2018). Since career development is rooted in processes in early childhood, this work will make important contributions to society as well as existing literature. First, it will contribute to the understanding of the predictors of communal occupational aspirations and communal orientation and thus might enable us to draw conclusions about how to foster boys’ interest in communal roles. Second, previous studies have rarely examined predictors of occupational aspirations in early childhood (see Fulcher et al., 2008; Fulcher, 2011 for exceptions). The present study adds to existing literature by focusing on children aged 4 ½ to 13 years old. Finally, the present work investigates career development in an interesting cultural context, namely Norway, one of the most gender egalitarian countries in the world (World Economic Forum, 2020).

### The Development of Occupational Aspirations

Over the past decades, several theories have emerged to describe the development of occupational aspirations (Ginzberg, 1952; Roe, 1957; Havighurst, 1964; Gottfredson, 1981). In 1952, Ginzberg published a general theory of occupational choice in which he divided childhood into two periods. In the first period (fantasy choice), children explore a wide range of different occupations, within a framework of their parents’ occupations and suggestions. In the second period (tentative choice), children aspire toward occupations based on their interests. In 1957, Roe formulated a theory of occupational choice that focused on quality of early family experiences (Trice et al., 1995). Havighurst developed a theory in 1964 that divided childhood into two main periods (identifying with the worker and acquiring basic habits/skills) in which the worker observed by children is often a parent. In 1981, Gottfredson published a theory based on a four-stage model (see Trice et al., 1995 for a short overview of all four abovementioned theories). This theory states that children first recognize adults’ occupational roles (1. Stage: from 3 to 5 years) and in a next stage (2. Stage: from 6 to 8 years) categorize occupational roles based on how appropriate they are for each gender—which most likely will be based on parental occupations. Taken together, although the outlined theories of occupational choice differ in many regards, they all highlight the important role of parents in the development of young children’s occupational aspirations. For this reason, in the present paper we focus on parental influence on children’s occupational aspirations and integrate these earlier theories with recent theories on how parents can act as role models for their young children.

### Parents as Role Models for Their Children

Social cognitive theory by Albert Bandura (Bussey and Bandura, 1999) posits that direct tuition, modeling, and enactive experience shape children’s gender development. Bussey and Bandura state that direct tutoring is a way to inform children about different types of behavior and how these behaviors can be linked to their gender. They further propose that a large amount of gender-linked information is exemplified by models. According to the motivational role modeling theory by Morgenroth et al. (2015), role models have three functions: they (1) act as behavioral models, (2) represent what is possible, and (3) are inspirational. This theory integrates expectancy-value models of motivation with the role model literature. When it comes to role models as behavioral models, Morgenroth et al. (2015) argue that someone who is motivated to pursue a goal will look to behavioral role models who demonstrate how to achieve this goal. This applies very well to children as they often look to their parents for help and parents show children how to achieve their goals. Second, Morgenroth et al. (2015) describe a role model as a representation of the possible (i.e., showing that something is possible to achieve). Lastly, when it comes to role models being inspirational (which Morgenroth et al. (2015), define as something that someone will see as desirable and worth working for), we again argue that parents may play a role by achieving highly desirable goals of their own. All three aspects of the theory may sometimes, but not always apply for parents and their children and will most likely vary with age and context.

Based on these theoretical approaches, the following work will focus on three ways in which parents may influence their children’s occupational aspirations and communal orientation: (1) through modeling of specific careers, (2) through the communication of gender-related attitudes, and (3) through modeling behaviors at home. Thus, in the next section we will summarize existing research on how parents influence their children’s occupational aspirations and orientation.

### Do Parents’ Occupations Affect Children’s Occupational Aspirations and (Communal) Orientation?

One of the main functions of a role model is to be a behavioral model (Morgenroth et al., 2015). One way parents influence their children’s occupational aspirations is through modeling behavior in work-related settings (and talking about their work at home, Parks-Stamm et al., 2020), thereby making potential occupational roles approachable for children. In line with this theoretical assumption, early research found that young women and men tend to aspire toward similar careers as their fathers (e.g., Holland, 1962; Werts and Watley, 1972). More recent contributions have investigated the influence of mothers’ occupation on young people’s occupational aspirations. This research has consistently shown that mothers’ employment outside the house, and the stereotypicality of their occupation is related to their children’s career-related attitudes and the traditionalism of their occupational aspirations (Burlin, 1976; Barak et al., 1991; Castellino et al., 1998; Riggio and Desrochers, 2006; van Putten et al., 2008; Fulcher and Coyle, 2011). For example, a study from the Netherlands showed that women who had mothers who worked in paid labor tended to work more when they grew up compared to women whose mothers were homemakers (van Putten et al., 2008). Research also shows that parents’ work participation influences their young adult children’s (aged 17 to 22) plans for work participation, with parents of the same sex as the child serving as the main role models (Wiese and Freund, 2011). Taken together, these studies show that parents’–and especially mothers’–occupations affect their children’s occupational aspirations in adolescence and young adulthood; however, less is known about this relationship in early childhood.

Furthermore, not only do parents influence their children’s occupational aspirations, they can also influence different aspects of children’s self-concept (e.g., Khan et al., 2017). When parents display different behaviors related to their occupational role, they can influence their children’s communal and agentic orientation. A study by Rainy and Borders (1997) found that characteristics of the mother (educational status, employment status, gender role attitudes, and agentic traits) were strong predictors of their daughters’ (aged 12 to 15 years) gender role attitudes. In addition, a study by Alessandri (1992) found that maternal employment among single parents positively affected children’s (aged 10–12 years) self-esteem. Girls with mothers employed full-time perceived themselves to have greater scholastic competence relative to boys and to boys and girls with mothers employed part-time. However, Rollins and White (1982) found no relationship between children’s (aged 10–14 years) self-concept (physical, moral, personal, family, social, and academic/work) and their mothers’ career choice (traditional, dual work or dual career). Therefore, previous research remains inconclusive concerning the role parents’ occupation plays for children’s (gender) related self-concept.

### Do Parents’ Gender Attitudes Affect Children’s Occupational Aspirations and (Communal) Orientation?

In addition to modeling behavior outside the home, parents express their gender attitudes as well as general attitudes directly to their children. Several studies have shown that parents’ gender attitudes align with their children’s gender roles and occupational aspirations (Starrels, 1992; Crouter et al., 2007; Fulcher et al., 2008; Fulcher, 2011; Croft et al., 2014). For example, in a study of 7- to 12-year-old children, Fulcher (2011) showed that mothers with more traditional attitudes had children with more self-efficacy for occupations in traditional domains. Crouter et al. (2007) investigated the development of gender attitudes in a longitudinal study across middle childhood and adolescence (aged 7 to 19 years). They found that girls whose parents held more traditional attitudes maintained more traditional attitudes over time compared with girls whose parents held less traditional attitudes. In sum, the research indicates that parents’ gender attitudes influence their children’s occupational aspirations (see also Halimi et al., 2016, for a review on the relationship between children’s gender role attitudes and individual, home, and school characteristics).

Parents’ gender attitudes can also affect children’s communal and agentic orientation. A study by Davis and Wills (2010) investigated how mothers’ and fathers’ gender attitudes influence their adolescent children (aged 14 and older). They found that when adolescents had a parent with gender egalitarian attitudes, they were more likely to be gender egalitarian themselves. A meta-analysis by Tenenbaum and Leaper (2002) investigated the relationship between parents’ gender schema and their children’s gender-related cognitions. The meta-analyses included research that had investigated this question in a broad age range from 2 years to 27 years. Children’s measures included children’s gender-concept, gender-related interests and preferences, and work-related attitudes and interests. They found that parents’ gender schemas were related to various types of their children’s orientation, which is a part of their gender self-concept. These results, however, were mostly based on correlational findings, so causal inferences could only be made with caution. Still, there is reason to think that parents do influence their children’s attitudes and particularly their gender-related attitudes in various ways (Tenenbaum and Leaper, 2002).

### Do Parents’ Share of Housework and Child Care Affect Children’s Occupational Aspirations and (Communal) Orientation?

Parents are also the primary models for their children when it comes to domestic work including housework and child care (see Coltrane, 2004, for a review on research on housework). Past research has investigated the effects of parents’ share of housework and child care on children’s occupational aspirations and their expectations about their future roles in the family and at work. This research shows that parents’ division of household tasks influences their children’s gender-role development (Cunningham, 2001; Deutsch et al., 2001; Crouter et al., 2007). Croft et al. (2014), for example, found that mothers who identified with traditional roles at home had children (aged 7 to 13 years) who imagined themselves in gender-stereotypical roles in the future. In addition, they found that when daughters had fathers who endorsed a more egalitarian distribution of housework, daughters expressed a greater interest in working outside of the home and in less stereotypical occupations. Another study found that in families where parents held liberal attitudes toward gender and had a more egalitarian division of labor, the children (aged 4 to 6 years) were more flexible in their own gender stereotypes and occupational aspirations (Fulcher et al., 2008). Thus, studies suggest that parents’ division of housework and gender role attitudes influence children’s aspirations not only to work outside of the home, but also to pursue less gender-stereotypical occupations.

In addition, parents’ division of housework and child care can affect children’s communal and agentic orientations. Research has shown that the more women are involved in male-dominated work (such as farm and ranch work), the more agentic (vs. communal) their value orientation becomes (Smyth et al., 2018). Thus, it is reasonable to anticipate that parents can influence their children in the same direction by their modeling of unpaid labor. This is in line with previous empirical research. For example, in a study with 10- and 11-year-old children, the researchers found that when fathers contributed more to driving children and arranging activities for them, the children endorsed a less stereotypical view of the family (Deutsch et al., 2001). Interestingly, it appeared that the effect of father’s participation in parenting on children’s stereotypes depended more on how fathers contributed rather than the amount of contribution. Taken together, studies suggest that the division of housework and child care can have an impact on children’s communal and agentic orientation and views of the family.

### The Present Research

The present research was conducted in Norway. Despite Norway’s high ranking in global gender equality indices and Norwegian women’s equal representation in higher education, the Norwegian labor market remains highly gender-segregated (SSB, 2016; Bufdir, 2019). For example, only about 10% of the pedagogical staff in childcare centers in Norway are male (Kristiansen et al., 2020). The number of men entering female-dominated education programs in Norwegian high schools has only increased slightly from 2010 to 2018 (SSB, 2019). For this reason, it seems particularly relevant to investigate how parents affect young children’s communal occupational aspirations and communal orientation in a very gender egalitarian country such as Norway. The present study adds to the literature by investigating the effects of parental influence on children’s occupational aspirations and communal orientation in a specific cultural context. Most previous research has been conducted in the United States (Rollins and White, 1982; Rainy and Borders, 1997; Castellino et al., 1998; Crouter et al., 2007; Davis and Wills, 2010; Fulcher, 2011). Therefore, with the present work we investigate the influence of the stereotypicality of mothers’ occupation, their gender attitudes, and their share of child care on children’s occupational aspirations in a highly gender egalitarian societal context to increase the understanding of how the societal context might influence the development of communal occupational aspirations and communal orientation.

In line with other researchers (e.g., Gottfredson, 1981; Hartung et al., 2005), we argue that early to middle childhood is a crucial period in which children start to develop occupational interests and aspirations, continuing throughout adolescence. For this reason, we conducted two studies as part of two larger research projects. In Study 1, we recruited young children aged 4 ½ to 6 years. In Study 2, we recruited elementary school children who were between 6 and 13 years old. The second study was part of a project that investigated the effects of COVID-19 on parents’ and children’s well-being (Martiny et al., 2021; Thorsteinsen et al., 2021) and also investigated the development of gender roles, gender stereotypes, and occupational aspirations in elementary school children.

Based on data from these two projects, we aimed to test the following research questions (preregistered on Open Science Framework: osf.io/47qh5).^1^ Concerning the relationship between the stereotypicality of mothers’ occupations and children’s aspirations, we predicted that children with mothers working in non-stereotypically female occupations will aspire less toward communal roles than children with mothers working in stereotypically female occupations (H1). Furthermore, we explored whether the association between the mother’s occupation and the child’s occupational aspirations was moderated by the gender (RQ2) and age (RQ3) of the child and tested the influence of parents’ gender attitudes and share of child care on young children’s communal occupational aspirations (RQ4). We also explored whether the associations between parents’ gender attitudes and children’s communal occupational aspirations were moderated by child gender (RQ5) and age (RQ6). We did not formulate directional hypotheses for RQ2–RQ6, as earlier research on these effects in this age-group is inconsistent, but studies have found stronger effects for older children (Tenenbaum and Leaper, 2002). Lastly, we explored the relationship between the stereotypicality of mothers’ occupation, gender attitudes, and share of child care on children’s communal orientation.

### Study 1: Childcare Center Study

This study was part of a larger project that investigated the development of gender roles, gender stereotypes, and occupational aspirations in young children (aged 4 ½ to 6 years) in Northern Norway. Data were collected in 2018.

## Materials and Methods

### Pilot Test

Before data collection, four pilot studies were conducted. Two pilot studies were conducted with adults, and the other two with young children. In the first pilot study, Norwegian adults (*N* = 28) were asked to report gender stereotypes for different jobs (e.g., “What % of kindergarten teachers in Norway are male?^2^”). The participants answered on a 100-point Likert scale ranging from 0% men to 100% men. The same group was then asked to report gender stereotypes for behaviors (i.e., “I associate comforting others with…”). Answers were reported on a 7-point Likert scale ranging from “Only women” (scored as 1) to “Only men” (scored as 7). In the second pilot, Norwegian adults (*N* = 37) were provided with definitions of communion and agency and were asked to identify the degree to which the selected occupations (based on the first pilot study) were perceived as communal and stereotypically female in a Norwegian context. Based on the results of these two pilot studies, the most prototypical communal occupations and behaviors were selected for the main study. A third and fourth pilot study was run with young children of the targeted age-group (four to six years). The aim of the third pilot was to assess the children’s (*N* = 8) ability to understand and engage with the study material. The fourth pilot study (*N* = 8) assessed the study length and children’s ability to concentrate during participation. After these two additional pilot tests, the study material was adjusted accordingly. For more details on the pilot studies, see Olsson (2021).

### Ethics

The project was registered with the Norwegian Centre for Research Data (NSD) and received ethical approval from the internal board of research ethics at the first author’s institution.

### Participants

Participants were recruited from public childcare centers in the entire county. After approval from the administrative/pedagogical leaders, parents with children in the targeted age-group (4 ½–6 years) were invited to participate. We recruited 177 children attending childcare centers in a small university town and up to two hours away, in Northern Norway. We excluded participants who were younger than the targeted sample (*n* = 18), withdrew their consent during testing (*n* = 7), did not follow instructions (*n* = 1), or had technical issues (*n* = 3). After exclusion, the final sample of children consisted of 159 children (84 boys and 75 girls) between the age of 54 and 75 months.

After the children had completed the study, children’s parents were invited to fill in a questionnaire. A total of 99 parents (80 mothers and 19 fathers) completed the questionnaire and returned it to the research team. Due to the low number of participating fathers, we decided to exclude them from the analyses. We were also unable to match eight of the mothers’ answers to their children’s. In the final sample of 72 mother–child dyads, children’s age ranged from four years and six months to six years and three months (*M* = 66.22 months, *SD* = 4.81; 37 boys and 35 girls). Mothers reported a mean age of 35.21 years (*SD* = 4.93; age missing for one mother).^3^

### Sensitivity Analyses

We conducted a *post hoc* sensitivity analyses with G^*^ Power (Faul et al., 2007) to explore the size of effects we were able to detect given a power of 0.95. With the current sample (*N* = 72) and three measures, we were able to detect an effect size of *f*^2^ = 0.15, which is considered a small effect (Faul et al., 2007).

### Recruitment and Procedure

Parents signed informed consent forms. Children were asked to give oral consent. Children were allocated to groups of up to four, with one male and one female experimenter. One experimenter explained the study, asked questions, and instructed the children in how to record their answers on a tablet. A second experimenter took notes during testing and assisted the children if necessary. During the testing, a teacher from the childcare center was present to assist with the children if necessary. After the testing of the children was completed, children’s parents received an invitation to fill in the parent questionnaire.

### Measures

All measures were in Norwegian. The measures reported in this paper can be found in English and Norwegian in the Supplemental Materials, which includes the visualizations of the scales used in the children’s part of the study.

#### Children’s Measures

##### Occupational Aspirations

Communal occupational aspirations were measured with three items. The experimenter read the following text to the children:

“I can imagine that you have thought about what you want to be when you grow up. When I went to kindergarten and thought about what I wanted to be when I grew up, I wanted to be so many things, not just one thing. I will now show you a few images of people who have different jobs. Although you might have decided what job you want to do later in life, I want you to tell me how much you would like to do *this* job” (for full description of how each occupation was described, see in the Supplementary Materials).

The children answered these questions for three communal roles (nurse, teacher at a childcare center, and stay-at-home parent) on a 3-point Likert scale ranging from “Not at all,” “Little”, to “A lot” by pressing smileys on a tablet. The first answering option was illustrated by a sad smiley, the second option with a neutral smiley, and the third with a happy smiley. The reliability of the scale was satisfactory (*α* = 0.61).^4^

##### Communal Orientation

We used the following four items to assess children’s communal orientation (i.e., help others who are upset, be close to others, hug others, and comfort others who are sad). The experimenter read the following text to the children:

“I will now read short stories about some children I know. It is your job to tell me whether this child sounds like you. I know a child who really, really likes to hug others and this child always gives hugs to other children. Does this sound like you?”

The children answered this on a 3-point Likert scale ranging from “Not at all,” “Little”, to “A lot.” The same visualization as for occupational aspirations was used. The reliability of the scale was good (*α* = 0.70).

#### Parents’ Measures

##### Mothers’ Occupation

In an open-ended question, participants were asked to report their current occupation. After data collection, the occupations were categorized into four categories—stereotypical/female-dominated, non-stereotypical/male-dominated, neutral, and unclassifiable occupations. We used information from the central statistics office in Norway (Utdanning, 2020) and classified the occupations into the following categories based on the following information: stereotypical/female-dominated (more than 65% of the workers in this domain are women), non-stereotypical/male-dominated (more than 65% of the workers in this domain are men), neutral (all occupations that neither belonged to category one or two) or unclassifiable. The inter-rater reliability of two independent raters was high (*κ* = 0.100). Of all occupations, 6.41% could not be classified because they lacked detailed information (e.g., assistant). Thirty-six occupations were assigned to the neutral category, thirty-two to the stereotypically female, and six to the non-stereotypically female category. Due to the non-stereotypical female category being so small, it was merged with the neutral category. The final coding consisted of two categories: stereotypically female occupations (*n* = 28) and non-stereotypically female occupations (*n* = 39). We dummy-coded the occupations (non-stereotypically female = 0, stereotypically female = 1).

##### Gender Attitudes

We measured gender attitudes with six items (selected from a scale developed by Beere et al., 1984). These included statements such as “Women should have the same chances as men to be leaders at work.” Parents answered how much they disagreed or agreed with the statements on a 7-point Likert scale ranging from 1 = “Strongly disagree” to 7 = “Strongly agree”. This measure showed a high reliability (*α* = 0.83).

##### Gender Essentialist Beliefs

We measured parents’ gender essentialist beliefs with 13 items with questions related to parenting (Gaunt, 2006) and work (selected from a scale developed by Beere et al., 1984). This included statements such as “Mothers are instinctively better caretakers than fathers” and “Men are naturally more inclined to do well in leader-positions.” The parents answered how much they disagreed or agreed with the statements on a 7-point Likert scale ranging from 1 = “Strongly disagree” to 7 = “Strongly agree”. This measure showed a high reliability (*α* = 0.87).

##### Share of Child Care

We assessed the parents’ division of child care with four items based on a self-developed scale. The items related to child care included items like “How much of the child care (taking care of the kids at home) do you/your partner do?” Parents answered these questions on a 7-point scale from 1 “I do everything” to 7 “My partner does everything.” There was also an option to choose “I do not have a partner” (coded as missing values in following analyses). The variable was recorded so that it ranged from 1 = “My partner does everything” to 7 = “I do everything”. The scale showed a high reliability (α = 0.84).^5^

##### Demographics

Parents were asked to categorize their income (into one of five income brackets ranging from NOK 0–320,000 to NOK more than 2,000,000) and report whether the child was bilingual or not. Parents were also asked to provide information about their child’s age, handedness, living situation, and number of siblings, the gender of their child’s teacher at the childcare center, and their own use of gender labels at home. Parents additionally provided demographic information (with respect to their immigrant background, level of education, and work hours per week) about themselves and (if applicable) about their partner.^6^

## Results

Means, standard deviations, and correlations of all variables can be found in Table 1. As can be seen in Table 1, there was a significant correlation between mothers’ occupation and children’s communal orientation, *r* = 0.25, *p* = 0.043. In addition, there was a significant correlation between children’s communal occupational aspirations and children’s communal orientation, *r* = 0.26, *p* = 0.025.

**Table 1:** Descriptive statistics and correlations for all relevant variables for the childcare center children.

	*M*	*SD*	*n*	1	2	3	4	5	6	7	8	9	10
1. Mothers ‘occupation	0.42	0.50	67	1									
2. Childs age	66.22	4.81	72	−0.03	1								
3. Childs gender	1.49	0.50	72	−0.07	0.13	1							
4. Childs bilingualism	1.17	0.38	72	−0.19	−0.09	0.16	1						
5. Children’s occupational aspirations	1.92	0.65	72	0.23^#^	−0.06	0.08	−0.02	1					
6. Children’s communal orientation	2.42	0.53	72	0.25^*^	0.12	0.09	−0.13	0.26^*^	1				
7. Parents share of childcare	4.45	0.60	70	−0.03	−0.06	0.05	−0.03	−0.02	0.02	1			
8. Parents gender attitudes	6.74	0.51	72	−0.24^#^	−0.07	0.09	−0.14	−0.00	0.01	−0.22^#^	1		
9. Parents essentialist beliefs	2.98	1.05	72	0.26^*^	0.15	0.11	−0.09	−0.07	0.29^*^	0.19	−0.35^**^	1	
10. Income	3.10	0.70	71	−0.19	0.29^*^	0.06	−0.39^**^	−0.06	0.08	−0.14	0.34^**^	−0.02	1

# *p<0.10*;

* *p < 0.05*;

** *p < 0.01*;

*Child gender (1 = boy, 2 = girl), bilingualism (1 = only Norwegian, 2 = bilingual), child age is reported in months*.

### Does the Stereotypicality of Mothers’ Occupation Predict Children’s Communal Occupational Aspirations?

First, we tested the main hypothesis, which is that children with mothers working in stereotypically female occupations will aspire more toward communal roles than children with mothers working in non-stereotypically female occupations. We used a linear regression analyses to test this hypothesis and controlled for child’s gender and age. As can be seen in Table 2, mothers’ occupation predicted children’s communal occupational aspirations, *B* = 0.31, *p* = 0.051. This means, in line with our hypothesis, that children with mothers working in stereotypically female occupations reported higher communal occupational aspirations (*M* = 2.06, *SD* = 0.65) than children with mothers working in non-stereotypically female occupations (*M* = 1.76, *SD* = 0.63). Both covariates were non-significant.^7^

**Table 2:** Linear regression for the effects of mothers’ occupation on childcare center children’s communal occupational aspirations while controlling for child gender and age.

	*B*	SE(B)	*β*	*t*	*R* ^2^	*p*
Child gender	0.24	0.16	0.19	1.56	0.09	0.125
Child age	−0.01	0.02	−0.06	−0.48		0.631
Mothers’ occupation	0.31	0.16	0.24	1.99		0.051

Next, we tested whether the child’s gender and age moderated the main effect of the mothers’ occupation. In order to test this, we conducted a linear regression analysis including the main effect of mother’s occupation, child gender, child age, and their two-way interactions. The effects of all two-way interactions were non-significant (all *p*s > 0.489). See Supplementary Table 6.

### Additional Exploratory Analyses

Next, we aimed to explore whether mothers’ share of child care and their gender attitudes related to children’s communal occupational aspirations. Mothers’ share of child care was z-standardized and used as an individual predictor of children’s communal occupational aspirations in a linear regression with child’s gender and age as main effects. The results showed no significant effect in share of child care predicting the communal occupational aspirations (*p* = 0.798). The effect of mothers’ share of child care on children’s communal occupational aspirations was not moderated by child gender (*p* = 0.885), but the interaction between mothers’ share of child care and child age approached significance (*p* = 0.051)^8^. In order to unpack the interaction between mothers share of child care and child age, interaction, we used Hayes (2018) PROCESS 4 moderation analyses with Model 1 to conduct the simple slopes analyses. We found no significant relationship for either of the age-groups (*ps* > 0.500). In addition to this, children’s communal occupational aspirations was not significantly related to mothers’ general gender attitudes (p = 0.603) or their essentialist beliefs (p = 0.447). The effect of mothers’ general gender attitudes on children’s communal occupational aspirations was not moderated by child gender or age (all *ps* > 0.464) nor was the effect of mothers essentiliast beliefs moderated by child gender or age (all ps > 0.099).

Finally, we tested the effects of mothers’ occupation on children’s communal orientation. Following the procedure outlined for the main hypotheses, we conducted a linear regression analysis with mothers’ occupation as predictor and children’s communal orientation as outcome, again controlling for child gender and age. As can be seen in Table 3, mothers’ occupation predicted children’s communal orientation, *B* = 0.27, *p* = 0.037. This means that children with mothers working in stereotypically female occupations reported higher on communal orientation (*M* = 2.61, *SD* = 0.48) than children with mothers working in non-stereotypically female occupations (*M* = 2.35, *SD* = 0.53). Both covariates (main effects and interactions) were non-significant.^9^ The effect was not moderated by child gender or child age (all *ps* < 0.347)

**Table 3:** Linear regression for the effects of mothers’ occupation on childcare center children’s communal orientation while controlling for child gender and age.

	*B*	SE(B)	*β*	*R* ^2^	*t*	*p*
Child gender	0.12	0.13	0.11	0.08	0.92	0.359
Child age	0.01	0.01	0.07		0.60	0.554
Mothers’ occupation	0.27	0.13	0.26		2.13	0.037

## Discussion

The aim of Study 1 was to investigate the relation between mothers’ occupation, gender attitudes, and share of child care on children’s communal occupational aspirations and communal orientation. In line with previous research (e.g., Barak et al., 1991), the gender stereotypicality of mothers’ occupation seems to influence young children’s occupational aspirations. Mothers working in stereotypically feminine occupations had children who reported higher aspirations toward communal occupations compared to children whose mothers worked in non-stereotypically feminine occupations. In addition, the stereotypicality of mothers’ occupation was significantly related to children’s communal orientation. This means that children with mothers working in stereotypically female occupations reported higher communal orientation compared to children with mothers working in non-stereotypically female occupations. Taken together, the results of this study provide support for the notion that mothers function as role models both for children’s communal occupational aspirations and for children’s communal orientation in early childhood.

### Study 2: Elementary School Study

In a second study, we explored whether these same patterns would be found in a sample of older children attending elementary school. This study was part of a larger project that investigated the consequences of the COVID-19 pandemic on parents and elementary school-aged children. Data were collected in June and July 2020, with an additional data collection in November and December 2020.

## Materials and Mehods

### Pilot Test

Because the data collection took place close to the school’s summer break, we had limited time to pilot-test our questionnaire. The children’s questionnaire was therefore only pilot-tested with two children. They were of the targeted age range, with one girl in second grade (seven years, and 10 months) and one boy in third grade (eight years and six months). The questionnaire was modified after receiving their comments.

### Ethics

The project was registered with the Norwegian Centre for Research Data (NSD) and received ethical approval from the internal board of research ethics at the first author’s institution.

### Participants

In this study, we recruited 98 children attending elementary schools in Norway. In addition to the children, 273 parents filled in a questionnaire.^10^ Five children were excluded either because we were unable to match them to their parent’s questionnaire (*n* = 3), or because they indicated that they did not understand the questionnaire (*n* = 2). In the remaining parent–child dyads, only six of the parent questionnaires were completed by fathers. Because of this low number, they were excluded from further analyses. Following these exclusions, the sample consisted of 87 mother–child dyads. We recruited more participants between November and December 2020 (Sample 2) as part of the larger COVID-19 project. The participants in Sample 2 answered the same questions as the participants collected in June 2020 (Sample 1) and consisted of 25 children and 52 parents. Of the 52 parents, 43 women and eight men answered the questionnaire (one participant did not report their gender). Again, due to low number of fathers participating, they were excluded from further analyses. In the remaining mother–child dyads, only twenty mothers could be matched to their children, and one child reported not understanding the questionnaire, leaving 19 mother–child dyads. We combined the samples from June 2020 and from November/December 2020. The final sample consisted of 106 mother–child dyads. Children’s age ranged from six to 13 years (*M* = 113.58 months, *SD* = 21.49; 48 boys and 58 girls). Mothers reported an average age of 39 years (*SD* = 5.76).^11^

### Sensitivity Analyses

We conducted a *post hoc* sensitivity analyses with G^*^ Power (Faul et al., 2007) to investigate the size of effects we were able to detect given a power of 0.95. With the current sample (*N* = 106) and three measures, we were able to detect an effect size of *f*^2^ = 0.10 which is considered a small effect (Faul et al., 2007).

### Recruitment and Procedure

Study 2 was conducted as an online questionnaire. We recruited parents by contacting principals of elementary schools. The principals were asked to distribute an invitation e-mail with a link to the survey either directly to parents of the targeted children, or to teachers for further distribution to parents. The children were then indirectly recruited through their parents. After parents completed their questionnaire, they were sent an e-mail with the link to the children’s questionnaire. Parents were asked to assist their children with filling out the questionnaire if necessary.

Participating parents gave consent for themselves and their children. In addition, children gave consent in the online program before starting their questionnaire. The children’s consent form and questionnaire were adapted to make it easier to understand. In addition, audio files were included on every page of the children’s questionnaire (these can be accessed in Norwegian on OSF: https://osf.io/4frk2/), so that the children could choose to have each question and answer option read to them. To illustrate the scale points, we used visualization such as smileys (see Supplementary Material).

### Measures

All measures were in Norwegian. The measures reported in this paper can be found in English and Norwegian in the Supplementary Material.

#### Children’s Measures

##### Occupational Aspirations

To assess communal occupational aspirations, children read or heard the following information: “Now we would like to ask you about how much you would like to work in different jobs. You have probably thought about what you want to be when you grow up, and maybe you have decided what job you want to work in later. Either way, we would like you to try to picture how much you would like some different jobs now. Pick the answer that suits you best. How much would you like to be a [job] when you grow up?” (for full description of each occupation, see Supplementary Material).

The children answered these questions for three occupations (nurse, teacher at a childcare center, and stay-at-home parent) on a 5-point Likert scale ranging from 1 = “Not at all” to 5 = “Very much”. The scale was illustrated with smileys, ranging from a non-smiling face to a smiling face. Due to low reliability when including the stay-at-home parent item (*α* = 0.48), this item was excluded. The correlation of the two remaining items was satisfactory (*r* = 0.46, *p* ≤ 0.001).

##### Communal Orientation

Communal orientation was measured with three items. The children read or heard the following text: “Now we would like to ask you about what you like to do. Many people like to do different things, so there are no correct or wrong answers. No one from your school or your friends will know what you have answered.”

The children answered three communal orientation questions: “Do you like to help other children when they are in pain?,” “Do you like to be with other children?,” and “Do you like to comfort other children when they are sad?” The children answered on a 5-point Likert scale ranging from 1 = “Not at all” to 5 = “Very much”. The same illustrative smileys were used here as above. The reliability of the scale was high (*α* = 0.80).^12^

#### Parents’ Measures

In this study, we only used the essentliast beliefs scale to measure parents’ attitudes towards gender equality (*α* = 0.88). We used a 5-point Likert scale ranging from 1 = “Strongly disagree” to 5 = “Strongly agree” for essentialist beliefs. We assessed the parents’ division of child care using the same scale as in Study 1. Parents answered these questions on a 7-point scale from 1 “I do everything” to 7 “My partner does everything.” There was also an option to choose “I do not have a partner” (these were coded as missing values in the following analyses). This variable was recorded so that it was scored from 1 = “My partner does everything” to 7 = “I do everything.” The measure of share of child care showed a good reliability (*α* = 0.84).

##### Mothers’ Occupation

Participants answered an open-ended question asking them to type in their occupation. Answers were coded using the same procedure as in Study 1: stereotypically female (*n* = 53) and non-stereotypically female and neutral combined (*n* = 22), and unclassifiable (*n* = 12). The inter-rater reliability of two independent raters was high (κ = 0.100). Of all occupations, 14.72% from Sample 1 could not be classified because they were lacking detailed information (e.g., municipality). The same coding was used for the answers retrieved from participants in Sample 2: stereotypically female (*n* = 14) and non-stereotypically female and neutral combined (*n* = 4) and 1 unclassifiable.

##### Demographics

As in Study 1, we asked mothers about their income and whether their children were bilingual or not. Because we had different income variables at the two data collection time points, and the income categories were too different to combine into one variable, we ended up coding income as a binary variable: low income = 1 (< NOK 460,000 for Sample 1 and < NOK 500,000 for Sample 2) or not low income = 2 (>NOK 460,000 for Sample 1 or > NOK 500,000 for Sample 2). Parents also answered questions about their children’s age, the use of gender labels in the home, the child’s living situation, siblings, and if the child was in a risk group for COVID-19. Parents also answered additional demographic questions about their immigration background, relationship status, education level, workhours per week, municipality, perceived local infection rates of COVID-19, and if they were in a risk group for COVID-19. If the parent indicated that they had a partner, they also reported the partner’s age, gender, occupation, workhours per week, and immigration background.

## Results

Means, standard deviations, and correlations of all variables can be found in Table 4. A significant correlation was observed between child’s gender and communal occupational aspirations, *r* = 0.27, *p* = 0.005, and between child’s gender and communal orientation, *r* = 0.24, *p* = 0.015. The stereotypicality of mothers’ occupation and children’s communal occupational aspirations were not correlated.

**Table 4:** Descriptive statistics and correlations for all relevant measures for the elementary school children.

	M	SD	N	1	2	3	4	5	6	7	8	9
1. Mothers’ occupation	0.72	0.45	93	1								
2. Childs age	113.58	21.49	106	0.01	1							
3. Childs gender	1.55	0.50	106	−0.04	−0.05	1						
4. Childs bilingualism	1.20	0.40	106	0.03	0.29^**^	−0.12	1					
5. Children’s occupational aspirations	1.74	0.94	106	−0.01	−0.15	0.27^**^	−0.05	1				
6. Children’s communal orientation	4.35	0.72	106	−0.10	0.02	0.24^*^	−0.02	0.15	1			
7. Parents share of childcare	4.72	0.85	86	0.20	0.05	0.01	0.23^*^	0.06	0.16	1		
8. Parents essentialist beliefs	2.69	0.73	106	0.04	0.04	0.00	0.21^*^	0.15	0.08	0.06	1	
9. Income	1.55	0.50	106	−0.12	0.02	−0.07	−0.12	−0.07	0.02	−0.34^**^	−0.32^**^	1

*Child age is measured in months. Child gender (1 = boy, 2 = girl), bilingualism (1 = only Norwegian, 2 = bilingual), Income variable is combined from both timepoints into two categories*.

* *p < 0.05*;

** *p < 0.01*.

### Does the Stereotypicality of Mothers’ Occupation Predict Children’s Communal Occupational Aspirations?

Following the procedure outlined in the preregistration, we tested the main hypotheses the same way as in Study 1. We used a linear regression to test the effect of mothers’ occupation on children’s communal occupational aspirations and controlled for child’s gender and child age. We also controlled for whether data were collected at time point 1 (coded as 1) or time point 2 (coded as 2). As can be seen in Table 5, mothers’ occupation did not significantly predict children’s communal occupational aspirations, *B* = 0.01, *p* = 0.946, but child gender did, *B* = 0.44, *p* = 0.023, with girls (*M* = 1.97, *SD* = 1.06) aspiring more toward communal roles than boys (*M* = 1.46, *SD* = 0.69). The main effect of age was not significant.^13^

**Table 5:** Linear regression for the effects of mothers’ occupation on elementary school children’s communal occupational aspirations while controlling for child gender and age.

	*B*	SE(B)	*β*	*R* ^2^	*t*	*p*
Child gender	0.44	0.19	0.24	0.08	2.32	0.023
Child age	−0.01	0.00	−0.15		−1.39	0.168
Dataset	−0.09	0.24	−0.04		−0.37	0.711
Mothers’ occupation	0.01	0.21	0.01		0.07	0.946

*In dataset variable, 1 = Sample 1, 2 = Sample 2*.

Next, in line with the preregistration and Study 1, we tested whether child gender and age moderate the main effect of mothers’ occupation. To test this, we conducted a linear regression analysis including the main effects of mothers’ occupation and child gender and age as well as their two-way interactions. The effects of all two-way interactions were non-significant (*p*s > 0.216). See Supplementary Table 7.

### Additional Exploratory Analyses

For all additional analyses, we employed the same analytic strategy as in Study 1, unless otherwise stated. As outlined in the exploratory part in the preregistration (RQ4), we examined whether mothers’ share of child care and their essentialist beliefs were associated with children’s communal occupational aspirations. Mothers’ share of child care showed no effect in predicting children’s communal occupational aspirations (*p* = 0.527), but child gender did (*p* = 0.029). The relationship between share of child care and communal occupational aspirations was not moderated by child’s gender or child age (*ps* > 0.533). Children’s communal occupational aspirations were also not predicted by mothers’ essentialist beliefs (p = 0.287), but child gender did (*p* = 0.026). The relationship between mothers’ essentialist beliefs and communal occupational aspirations were also not moderated by child gender or child age (ps > 0.095).

Finally, we again tested the effects of mothers’ occupation on children’s communal orientation. We conducted a linear regression analysis with mothers’ occupation as predictor and children’s communal orientation as outcome, again controlling for child’s gender and age. The stereotypicality of mothers’ occupation did not predict children’s communal orientation, but child’s gender approached significance, *B* = 0.29, *p* = 0.061.^14^ Specifically, girls reported slightly higher communal orientation (*M* = 4.51, *SD* = 0.58) compared with boys (*M* = 4.17, *SD* = 0.83). Finally, we tested whether there was a moderating effect of child gender or age but found no significant effects (*ps* > 0.317).

## Discussion

The aim of the second study was to investigate mothers’ influence on children’s communal occupational aspirations and communal orientation in middle childhood. In contrast to our predictions and the results from Study 1, we did not find an effect of the stereotypicality of mothers’ occupation on children’s communal occupational aspirations, but only a main effect of child gender. In addition, the stereotypicality of mothers’ occupation was not related to children’s communal orientation and none of the other parent variables (e.g., mothers’ essentialist beliefs and share of child care) were related to children’s communal occupational aspirations or communal orientation. Taken together, this indicates that in middle childhood the influence of mothers as role models might be reduced as other factors come into play, or that there were too many methodological differences between Study 1 and Study 2 which make them difficult to compare.

## General Discussion

The present findings reveal that mothers’ occupation predicted 4½–6-year-olds’ communal occupational aspirations and their communal orientation. In other words, the children who had mothers working in stereotypical female occupations aspired more toward communal roles and reported higher communal orientation. Interestingly, we were not able to replicate this effect with elementary school children. This might indicate that in elementary school there is a change in mothers’ influence on their children’s gender-related cognitions. Specifically, we found that mothers’ occupation neither predicted 6–13-year-olds’ communal occupational aspirations nor their communal orientation. The results indicated that—for both the 4½–6-year-olds and the 6–13-year-olds—mothers’ gender attitudes, share of child care, and essentialist beliefs did not predict their children’s communal occupational aspirations.

The present findings have important theoretical and practical implications. First, in line with earlier empirical research (Barak et al., 1991; Fulcher, 2011), these findings indicate that children’s occupational aspirations start to develop at an early age. The findings suggest that the stereotypicality of mothers’ occupations affects young children’s communal occupational aspirations. Children who had mothers working in stereotypically female occupations aspired more toward communal occupations than children with mothers working in non-stereotypically female occupations. This indicates that mothers function as role models for their young children with regard to future occupational aspirations. Second, in line with the motivational theory of role modeling (Morgenroth et al., 2015) this could mean that mothers function as behavioral role models, as well as a representation of the possible. For young children, mothers might function as a representation of the possible by displaying what occupations and roles it is possible to achieve and in turn function as a role model for the children’s own occupational trajectory. This is also in line with the earlier theories of occupational choice where parents often are seen as an inspiration for future occupations and where family experiences influence the children’s occupational choice. Interestingly, we found not only the predicted effect of the stereotypicality of mothers’ occupation on children’s communal occupational aspirations, but also on their communal orientation. This means that children who had mothers working in stereotypical female occupations reported higher communal orientation, such as liking to help others. This again is in line with role modeling theories. When mothers are behavioral role models, they show children how to achieve a goal. If the child has a friend who is upset, a goal could be to comfort the friend. The mother might show them how to comfort their friend, which in turn may lead to the child learning and appreciating the (communal) behavior. Taken together, mothers who display communality in their occupations will likely value communality and thus reinforce communal behavior in their children.

The fact that we could not replicate the results of Study 1 in Study 2 might indicate that different factors affect children’s communal occupational aspirations at different times throughout childhood. Our findings therefore might indicate that the stereotypicality of mothers’ occupation does not affect children’s communal occupational aspirations or communal orientation in middle childhood. This interpretation is in line with developmental research that shows that as children get older, relationships and attitudes of peers increasingly influence their communal and agentic orientation and behaviors (i.e., Poteat and Anderson, 2012; Benish-Weisman et al., 2021). However, the fact that we did not replicate the results of Study 1 in Study 2 might also be due to other reasons such as methodological differences, and therefore, we need to interpret them with caution. Specifically, the two studies differed in how the data were collected, the measures we used, and the occupations included in the analyses. First, data from Study 1 were collected physically with experimenters visiting childcare centers and directly interacting with the children, whereas Study 2 was an online study. Second, in Study 1 we asked about three occupations (nurse, teacher at a childcare center, and stay-at-home parent). Although it is debatable whether stay-at-home parent is really an occupation, or simply a communal role, we decided to include it as we assumed it would be a communal role that young Norwegian children would easily understand, as parents in Norway are entitled to 49 weeks of fully compensated parental leave (NAV, 2021). However, due to low reliability among older children we had to exclude the stay-at-home parent item in Study 2. Thus, the scale for occupational aspirations in Study 2 consisted of two items instead of the intended three. The reason the complete scale did not work as well for Study 2 might be because older children have a better understanding of the difference between roles and occupations. In other words, they know that it is normal for parents in Norway to take parental leave before going back to full-time work and therefore understand that it should not be considered an occupation. In addition, as mentioned, data from Study 2 were collected as part of an online study, where some children indicated that they had help from their parents while others did not. This might have influenced the results, as children might have turned to their parents for answers, which might have created different conditions of participation for the children. This may particularly apply to the youngest children, as they might have especially depended on their parents when unsure how to answer. Thus, social desirability might have played a role in this study.

Another potential limitation of the present study is the fact that we only used three communal roles/occupations as central dependent measures, which were further reduced to two in Study 2 because of low reliability. As outlined earlier, we had pilot-tested these occupations and chosen them because they were seen as particularly representative of communal roles by Norwegian adults. Still, we need to acknowledge that for the three selected communal occupations, the communality of each role was confounded with the number of women working in this role. Thus, we cannot be sure whether the present effects were indeed due to the communality of the role or to the overrepresentation of women in the role. However, since we did not find gender-specific effects in Study 1 (no moderation by child gender), it seems likely that the present effects, namely that children with mothers’ working in stereotypically female occupations are more inclined to pursue communal roles, were indeed driven by the communality of the role and not by the desire to work in an occupation in which women (i.e., for girls in which people with the same gender) are overrepresented.

Finally, we did not have an equal distribution of mothers working in stereotypically female and non-stereotypically female occupations in both studies. The majority of occupations in Study 1 were non-stereotypical (54.2%), while in Study 2 the majority of occupations were stereotypically female (63.2%). A more equal distribution would be beneficial for future research. In addition, we sometimes had difficulties assigning occupations to one of the three categories (stereotypically-female, non-stereotypically female, and neutral) and therefore these occupations were assigned to the category “unclassifiable”. This was particularly difficult in Study 2 because the mothers in this study provided less precise information about their occupation. Finally, in addition to the limitations that have been outlined above related to Study 2, the data from Study 2 were collected after the national lockdown in Norway due to the COVID-19 pandemic. During the period of national lockdown, nurses had especially been praised in social media and the news (Mohammed et al., 2021; Turale and Nantsupawat, 2021). Along with the additional pressure experienced by teachers with the sudden change to online school, these changes in occupational perceptions could have influenced the older children’s aspirations toward these occupations.

Based on the differences between the results from Studies 1 and 2, future research needs to investigate in more detail whether parents’ influence on children’s occupational aspirations change from early to middle childhood by using larger samples and different methodology to test the generalizability of the present effects. In addition, a longitudinal design would contribute to understanding the stability of effects and would be able to capture change across development in childhood.

## Conclusion

The present research adds to the literature on predictors of young children’s occupational aspirations. Specifically, the findings demonstrate that the stereotypicality of mothers’ occupations is associated with their young children’s occupational aspirations toward communal careers and suggest that up to a certain age, parents—at least mothers—are important role models for their children. We are cautious when interpreting the lack of significant effects in Study 2, but the lack of support for mothers’ occupations, attitudes, and share of child care associated with elementary school children’s occupational aspirations and communal orientation might indicate that in middle childhood other factors exert more influence.

## Data Availability Statement

The raw data supporting the conclusions of this article is avaliable at: https://osf.io/4frk2/ under the folder “Does the stereotypicality of mothers’ occupation influence children’s occupational aspirations and communal orientation?”.

## Author Contributions

MITO and SEM conceptualized and designed Study 1. MK, KT, MO, and SEM conceptualized and designed Study 2. MO and KT organized the data. MK performed the statistical analysis. MK, SEM, and KT wrote sections of the manuscript. All authors contributed to editing the manuscript and approved the submitted version.

## Conflict of Interest

The authors declare that the research was conducted in the absence of any commercial or financial relationships that could be construed as a potential conflict of interest.

## Publisher’s Note

All claims expressed in this article are solely those of the authors and do not necessarily represent those of their affiliated organizations, or those of the publisher, the editors and the reviewers. Any product that may be evaluated in this article, or claim that may be made by its manufacturer, is not guaranteed or endorsed by the publisher.

## Footnotes

^1^Since the preregistration was written after the data from Study 1 were analyzed, only the hypotheses of Study 2 were preregistered. We initially planned to combine the data from Study 1 and Study 2 for analyses (see preregistration), but only later realized that this was not possible because the two studies differed in too many regards (different conditions of data collection, differences in the internal reliabilities of the scales, etc.) Thus, we deviated from the preregistered plan and conducted the analyses of Study 1 and Study 2 separately.

^2^Here we refer to kindergarten teachers as a part of the pedagogical staff in childcare centers.

^3^Of the participating mothers, none reported to be in a relationship with another woman. Three did not report their partner’s gender.

^4^In addition to asking about communal occupational aspirations, we asked the children about their aspirations towards agentic occupations (boss and police officer for Study 1 and firefighter, soldier, and pilot for Study 2) and their agentic orientation. Due to low reliabilities for these scales, they were not included in the analyses.

^5^In addition, we assessed parents’ division of housework. This was measured with items such as “Who does the dishes?” and “Who does the laundry?.” These measures are not included in this report.

^6^In Study 1 we also assessed children’s occupational gender stereotypes, implicit stereotypes (in an auditory Stroop task), role model qualities (concerning their teachers), agentic orientation, and agentic occupational aspirations.

^7^This main effect of mothers’ occupation on children’s communal occupational aspirations holds (*p* = 0.037) when income and bilingualism are added as additional covariates in the regression analyses.

^8^When including the interaction terms in the model, our predictor approached significance (*p* = 0.054), and child age became significant (*p* = 0.046). However, this does not appear to be a robust effect, and should be interpreted with caution.

^9^This main effect of mothers’ occupation on childcare center children’s communal orientation holds (*p* = 0.023) when income and bilingualism are added as additional covariates in the regression analyses.

^10^In the Norwegian educational system, elementary school is from 1^st^ to 7^th^ grade. Children start the year they turn six years old and finish the year they turn 13.

^11^Of the participating mothers, one indicated having a female partner and 17 did not report partner’s gender. Because the item assessing mothers’ share of child care did not refer to a male partner, we decided to include these participants in the analyses.

^12^In Study 2, we also assessed children’s perceived performance in school, well-being, emotions, experiences of home schooling during the pandemic, agentic orientation, descriptive gender stereotypes, and general attitudes toward school. These measures are not included in this report.

^13^This main effect of gender on elementary school children’s communal occupational aspirations holds (*p* = 0.028) when income and bilingualism are added as additional covariates in the regression analyses.

^14^The results slightly change when income and bilingualism are added as additional covariates in the regression analyses. Child gender still approaches significance, *p* = 0.068.
