# Supplementary material for: Does the Stereotypicality of Mothers’ Occupation Influence Children’s Communal Occupational Aspirations and Communal Orientation?
Source: Front Psychol. 2021 Dec 16;12:730859. doi: 10.3389/fpsyg.2021.730859 (PMC9231506; doi:10.3389/fpsyg.2021.730859)
Supplement: Supplementary file 1 [file Data_Sheet_1.docx]

***Supplementary Material***

In this section, we report the following additional information: A) a detailed overview of the original measures in the study, Tables 1 to 5, B) the full regression models for the main analyses.

1. **Overview of Measures**

Table 1 to 5 include variables, visualization, and Norwegian and English wording of the original items. The original audio files for Study 2 (Norwegian) can be found in the “Audio files”- folder under the OSF project folder (<https://osf.io/4frk2/> ). In addition to the measures reported here, in the children’s questionnaire in Study 2 we assessed: general attitudes towards the COVID-19 restrictions, well-being, emotions, experiences of home schooling, positive and negative emotions, agentic orientation, status of communal and agentic occupations, gender stereotypes for communal and agentic occupations and demographics. In the parents’ questionnaire we also assessed: retrospective measures of child well-being under normal circumstances and during the lockdown, changes at children’s school, parent well-being, stress, children’s attitudes toward school before, during and after lockdown, demographics about themselves, their child, and their partner, and at the end participants were given the opportunity to give additional information or comments. For Sample 2 in Study 2 we also assessed parents’ social interactions during lockdown and the previous month, and children’s screen time. In Study 1 we also assessed children’s occupational gender stereotypes, implicit stereotypes (in an auditory Stroop task), and asked questions about the children’s perception of one of their childcare center teachers. Additional parent measures for Study 1 includes demographics and importance of communal and agentic orientation.

***Parents’ Measures***

All parents’ measures reported here were the same for Study 1 and Study 2. In Study 1 the scale for gender attitudes and biological essentialist beliefs ranged from 1-5, and 1-7 for Study 2.

Table 1

*The Eight Items for Measuring Parents’ Share of Housework (four items) and Childcare (four items). Scale developed by Olsson, 2021.*

| Language | Item | Scale points | | | | | | |  |
| --- | --- | --- | --- | --- | --- | --- | --- | --- | --- |
| English | How much of the cooking do you/your partner do? | 1- I do everything | 2 | 3 | 4 | 5 | 6 | 7 – My partner does everything | 8- N/A – I don’t have a partner |
|  | How much of the house cleaning do you/your partner do? | 1- I do everything | 2 | 3 | 4 | 5 | 6 | 7 – My partner does everything | 8- N/A – I don’t have a partner |
|  | How much of the laundry do you/your partner do? | 1- I do everything | 2 | 3 | 4 | 5 | 6 | 7 – My partner does everything | 8- N/A – I don’t have a partner |
|  | How much of the dishes do you/your partner do? | 1- I do everything | 2 | 3 | 4 | 5 | 6 | 7 – My partner does everything | 8- N/A – I don’t have a partner |
|  | How much of the childcare (taking care of the kids at home) do you/your partner do? | 1- I do everything | 2 | 3 | 4 | 5 | 6 | 7 – My partner does everything | 8- N/A – I don’t have a partner |
|  | How much of the childcare (spending time with the kids) do you/your partner do? | 1- I do everything | 2 | 3 | 4 | 5 | 6 | 7 – My partner does everything | 8- N/A – I don’t have a partner |
|  | How much of the childcare (attending to their physical needs) do you/your partner do? | 1- I do everything | 2 | 3 | 4 | 5 | 6 | 7 – My partner does everything | 8- N/A – I don’t have a partner |
|  | How much of the childcare (attending to their emotional needs) do you/your partner do? | 1- I do everything | 2 | 3 | 4 | 5 | 6 | 7 – My partner does everything | 8- N/A – I don’t have a partner |
| Norwegian | Hvor mye av oppvasken hjemme hos dere tar henholdsvis du og din partner? | 1- Jeg tar alt | 2 | 3 | 4 | 5 | 6 | 7 – Partneren min tar alt | 8 – N/A – Jeg har ikke en partner |
|  | Hvor mye av husvasken gjør henholdsvis du og din partner? | 1- Jeg tar alt | 2 | 3 | 4 | 5 | 6 | 7 – Partneren min tar alt | 8 – N/A – Jeg har ikke en partner |
|  | Hvor mye at matlagingen gjør henholdsvis du og din partner? | 1- Jeg tar alt | 2 | 3 | 4 | 5 | 6 | 7 – Partneren min tar alt | 8 – N/A – Jeg har ikke en partner |
|  | Hvor mye av klesvasken gjør henholdsvis du og din partner? | 1- Jeg tar alt | 2 | 3 | 4 | 5 | 6 | 7 – Partneren min tar alt | 8 – N/A – Jeg har ikke en partner |
|  | Hvor mye av barnepasset ved å ta vare på barna hjemme tar henholdsvis du og din partner? | 1- Jeg tar alt | 2 | 3 | 4 | 5 | 6 | 7 – Partneren min tar alt | 8 – N/A – Jeg har ikke en partner |
|  | Hvor mye av barnepasset ved å tilbringe tid med barna tar henholdsvis du og din partner? | 1- Jeg tar alt | 2 | 3 | 4 | 5 | 6 | 7 – Partneren min tar alt | 8 – N/A – Jeg har ikke en partner |
|  | Hvor mye av barnepasset ved å oppfylle deres fysiske behov tar henholdsvis du og din partner? | 1- Jeg tar alt | 2 | 3 | 4 | 5 | 6 | 7 – Partneren min tar alt | 8 – N/A – Jeg har ikke en partner |
|  | Hvor mye av barnepasset ved å oppfylle barnas følelsesmessige behov tar henholdsvis du og din partner? | 1- Jeg tar alt | 2 | 3 | 4 | 5 | 6 | 7 – Partneren min tar alt | 8 – N/A – Jeg har ikke en partner |

Table 2

*Thirteen Items for Measuring Parents Biological Essentialist Beliefs about parenting (Gaunt, 2006) and working (Selected items from Beere et al., 1984).*

| Language | Item | Scale points | | | | | | |
| --- | --- | --- | --- | --- | --- | --- | --- | --- |
| English | There is no difference between men and women when it comes to taking care of children. | 1- Strongly disagree | 2 | 3 | 4 | 5 | 6 | 7 – Strongly agree |
|  | Women are instinctively better caretakers than fathers. | 1- Strongly disagree | 2 | 3 | 4 | 5 | 6 | 7 – Strongly agree |
|  | Mothers are naturally more sensitive to a baby’s feelings than fathers are. | 1- Strongly disagree | 2 | 3 | 4 | 5 | 6 | 7 – Strongly agree |
|  | Fathers have to learn what mothers are able to do naturally in terms of child care. | 1- Strongly disagree | 2 | 3 | 4 | 5 | 6 | 7 – Strongly agree |
|  | Men could nurture like women. | 1- Strongly disagree | 2 | 3 | 4 | 5 | 6 | 7 – Strongly agree |
|  | If they only wanted to, men could take care of children just as well as women. | 1- Strongly disagree | 2 | 3 | 4 | 5 | 6 | 7 – Strongly agree |
|  | Maternal instincts enable mothers to identify baby’s needs. | 1- Strongly disagree | 2 | 3 | 4 | 5 | 6 | 7 – Strongly agree |
|  | Women tend to pursue degrees in education and social work because they are naturally helpful and caring. | 1- Strongly disagree | 2 | 3 | 4 | 5 | 6 | 7 – Strongly agree |
|  | Women are naturally inclined to excel in jobs such as secretary, nurse, and teacher. | 1- Strongly disagree | 2 | 3 | 4 | 5 | 6 | 7 – Strongly agree |
|  | Women are naturally inclined to be more caring and nurturing. | 1- Strongly disagree | 2 | 3 | 4 | 5 | 6 | 7 – Strongly agree |
|  | Men have a tendency to take an education in science, technology, math and engineering because they are naturally gifted with these abilities. | 1- Strongly disagree | 2 | 3 | 4 | 5 | 6 | 7 – Strongly agree |
|  | Men, more so than women, are naturally inclined to be career oriented. | 1- Strongly disagree | 2 | 3 | 4 | 5 | 6 | 7 – Strongly agree |
|  | Men are naturally more inclined to do well in leader positions. | 1- Strongly disagree | 2 | 3 | 4 | 5 | 6 | 7 – Strongly agree |
| Norwegian | Det er ingen forskjell mellom menn og kvinner når det gjelder å kunne ta vare på barn. | 1- Sterkt uenig | 2 | 3 | 4 | 5 | 6 | 7 – Sterkt enig |
|  | Mødre er instinktivt bedre til å ta vare på barn. | 1- Sterkt uenig | 2 | 3 | 4 | 5 | 6 | 7 – Sterkt enig |
|  | Mødre er av natur mer sensitive til barns velvære enn fedre er. | 1- Sterkt uenig | 2 | 3 | 4 | 5 | 6 | 7 – Sterkt enig |
|  | Fedre må lære det mødre av natur er gode på når det gjelder å kunne ta vare på barn. | 1- Sterkt uenig | 2 | 3 | 4 | 5 | 6 | 7 – Sterkt enig |
|  | Menn kan vise omsorg for barn slik kvinner kan. | 1- Sterkt uenig | 2 | 3 | 4 | 5 | 6 | 7 – Sterkt enig |
|  | Hvis de bare vil, så kan menn ta vare på barn like bra som kvinner kan. | 1- Sterkt uenig | 2 | 3 | 4 | 5 | 6 | 7 – Sterkt enig |
|  | Morsinstinktet hjelper mødre å gjenkjenne barnas behov. | 1- Sterkt uenig | 2 | 3 | 4 | 5 | 6 | 7 – Sterkt enig |
|  | Kvinner har en tendens til å ta utdanning i sosialt arbeid fordi de av natur er hjelpsomme og omsorgsfulle. | 1- Sterkt uenig | 2 | 3 | 4 | 5 | 6 | 7 – Sterkt enig |
|  | Kvinner er av natur mer sannsynlige til å utmerke seg i jobber som sekretær, sykepleier og lærer. | 1- Sterkt uenig | 2 | 3 | 4 | 5 | 6 | 7 – Sterkt enig |
|  | Kvinner er av natur mer tilbøyelig til å være omsorgsfulle og pleiende. | 1- Sterkt uenig | 2 | 3 | 4 | 5 | 6 | 7 – Sterkt enig |
|  | Menn har en tendens til å ta utdanning innen vitenskap, teknologi, matematikk og ingeniør fordi de har naturlige evner i disse fagfeltene. | 1- Sterkt uenig | 2 | 3 | 4 | 5 | 6 | 7 – Sterkt enig |
|  | På grunn av forskjeller mellom menn og kvinner har menn en tendens til å være mer konkurrende og karrieredrevet enn kvinner. | 1- Sterkt uenig | 2 | 3 | 4 | 5 | 6 | 7 – Sterkt enig |
|  | Menn er av natur mer tilbøyelig til å utmerke seg i lederstillinger. | 1- Sterkt uenig | 2 | 3 | 4 | 5 | 6 | 7 – Sterkt enig |

Table 3

*Six Items Measuring Parents General Gender Attitudes (selected from a scale developed by Beere et al., 1984). This was only measured in Study 1 and Sample 1 of Study 2.*

| Language | Item | Scale points | | | | | | |
| --- | --- | --- | --- | --- | --- | --- | --- | --- |
| English | Women ought to have the same chances as men to be leaders at work. | 1- Strongly disagree | 2 | 3 | 4 | 5 | 6 | 7 – Strongly agree |
|  | Mothers and fathers should share the responsibility of taking their kids to the doctor or dentist. | 1- Strongly disagree | 2 | 3 | 4 | 5 | 6 | 7 – Strongly agree |
|  | Men and women should get equally paid for equal work. | 1- Strongly disagree | 2 | 3 | 4 | 5 | 6 | 7 – Strongly agree |
|  | A woman’s career should be just as important as a man’s career. | 1- Strongly disagree | 2 | 3 | 4 | 5 | 6 | 7 – Strongly agree |
|  | When both the husband and wife work outside of the home, housework should be equally shared. | 1- Strongly disagree | 2 | 3 | 4 | 5 | 6 | 7 – Strongly agree |
|  | A woman should have as much right to ask a man for a date as a man has to ask a woman for a date. | 1- Strongly disagree | 2 | 3 | 4 | 5 | 6 | 7 – Strongly agree |
| Norwegian | Kvinner burde ha de samme sjansene som menn til å være ledere på jobb. | 1- Sterkt uenig | 2 | 3 | 4 | 5 | 6 | 7 – Sterkt enig |
|  | Mødre og fedre burde dele ansvaret med å ta barna til legen eller tannlegen. | 1- Sterkt uenig | 2 | 3 | 4 | 5 | 6 | 7 – Sterkt enig |
|  | Menn og kvinner burde lønnes likt for likt arbeid. | 1- Sterkt uenig | 2 | 3 | 4 | 5 | 6 | 7 – Sterkt enig |
|  | En kvinnes karriere burde være like viktig som mannens. | 1- Sterkt uenig | 2 | 3 | 4 | 5 | 6 | 7 – Sterkt enig |
|  | Når både mannen og kvinner jobber utenfor hjemmet burde husarbeidet deles likt. | 1- Sterkt uenig | 2 | 3 | 4 | 5 | 6 | 7 – Sterkt enig |
|  | En kvinne burde ha like mye rett til å be en mann på stevnemøte som en mann har å be en kvinne. | 1- Sterkt uenig | 2 | 3 | 4 | 5 | 6 | 7 – Sterkt enig |

**Children’s Measures: Study 1**

*Communal Occupational Aspirations*

Instruction text:

English: “I can imagine that you have thought about what you want to be when you grow up. When I went to kindergarten and thought about what I wanted to be when I grew up, I wanted to be so many things, not just one thing. I will now show you a few images of people who have different jobs. Although you might have decided what job you want to do later in life, I want you to tell me how much you would like to do *this* job”

Experimenter shows image of [job]

1. [nurse] “What have we got here? Plasters and syringe. Who uses this? A Nurse who cares for people who are sick. Would you like to be a nurse when you grow up?”
2. [stay-at-home parent] “What have we got here? Someone who feeds a baby. Who does that? Someone who does not work but stays at home and looks after their baby instead. Would you like to stay at home and look after your baby when you grow up?”
3. [kindergarten teacher] “What have we got here? There are children here. Who looks after children? A kindergarten teacher. Would you like to be a kindergarten teacher when you grow up?”

Norwegian: “Jeg kan tenke meg at du har tenkt på hva du har lyst til å jobbe med når du blir stor. Da jeg gikk i barnehagen og tenkte på hva jeg ville bli når jeg ble stor, så hadde jeg lyst til å bli mange ting, ikke bare en ting. Nå skal jeg vise deg noen bilder av forskjellige jobber. Selv om du kanskje har bestemt deg for hvilken jobb du vil ha, vil jeg at du skal prøve å se for deg å ha denne jobben og fortelle meg hvor mye du hadde likt å ha denne jobben.”

1. “Hva er det vi har her? Noen som mater en baby. Hvem gjør dette? Noen som er hjemme og passer på babyen sin. Hvor mye har du lyst til å være hjemme og å passe på babyen din når du blir stor?”
2. “Hva er det vi har her? Det er barn her. Hvem passer på barn? En barnehagelærer. Hvor mye har du lyst til å bli en barnehagelærer når du blir stor?”
3. “Hva er det vi har her? Plaster og en sprøyte. Hvem bruker dette? En sykepleier som tar vare på de som er syke. Hvor mye har du lyst til å bli en sykepleier når du blir stor?”

**Images used for occupational measure:**


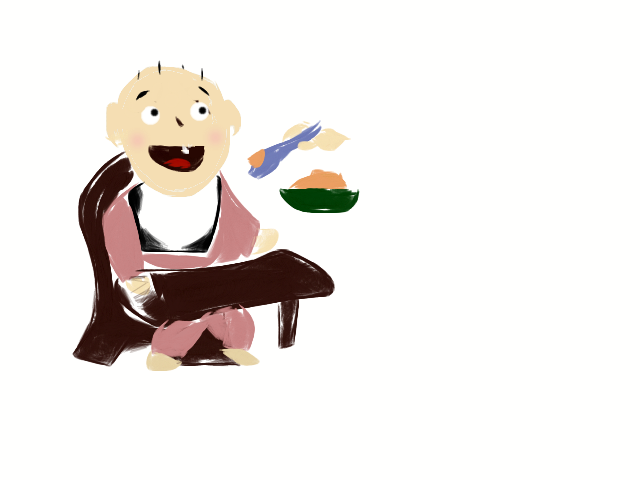
**Figure SM1**Stay-at-home parent.

**
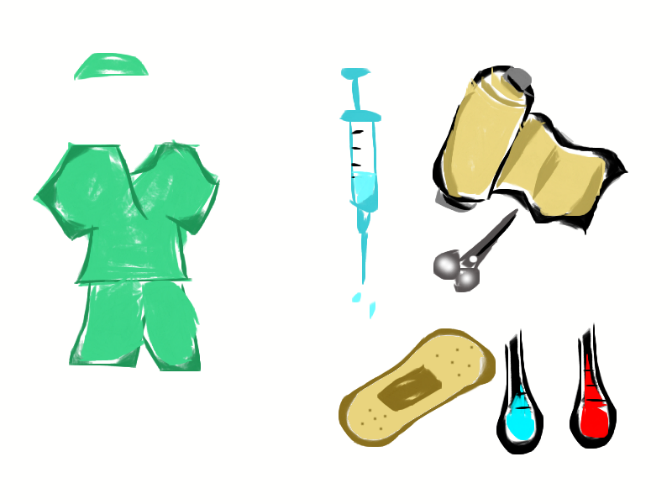
Figure SM2**Nurse.

**
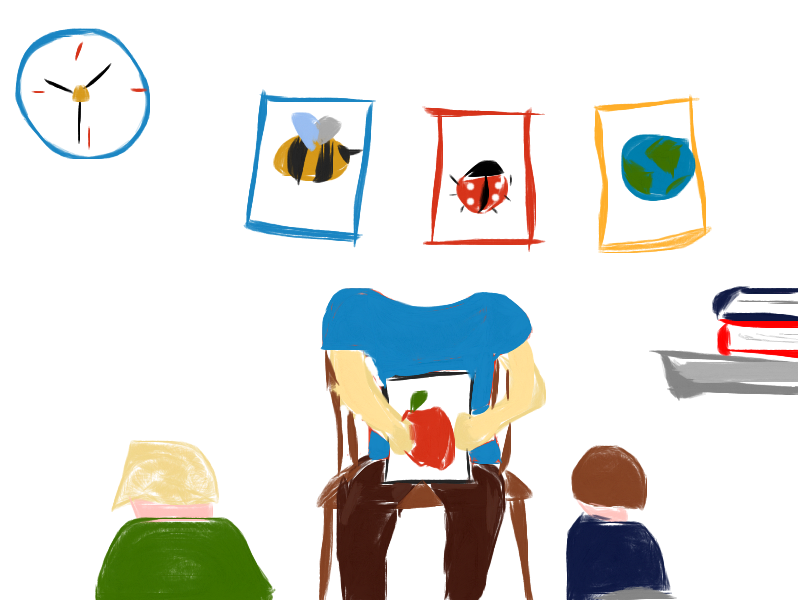
Figure SM3**Kindergarten Teacher.

**Figure SM4**
Illustrations used as the children’s answering options:

**
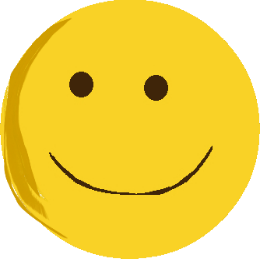

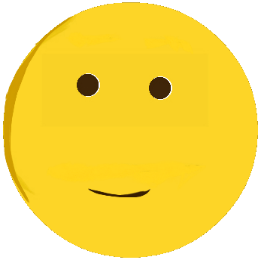

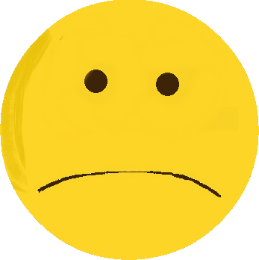
**

Scale range: Not at all, Little, A lot.

*Children’s communal orientation*

Instruction text:

English: “I will now read short stories for you about some children I know. It is your job to tell me whether this child sounds like you.”

1. “I know a child who tries to help other children, if they see that they are upset. Does this sound like you?”
2. “I know a child who really, really likes to be together with others and be close to others. Does this sound like you?”
3. “I know a child who really, really likes to hug others and this child always gives hugs to other children. Does this sound like you?”
4. “I know a child who always comforts others when they see that they are upset. Does this sound like you?”

Response options:

We used the same illustrations as in the occupational measures. Press on the face that does not smile if you disagree, press on the face with the little smile if you agree a little bit, or press on the face with the big smile if you agree a lot.

Norwegian: “Nå skal jeg lese noen korte fortellinger for dere om noen barn som jeg kjenner. Det jeg vil at du skal gjøre, er å si om dette barnet høres ut som deg”.

1. “Jeg vet om et barn som prøver å hjelpe, hvis de ser at et annet barn er trist eller lei seg. Pleier du å hjelpe andre barn som er lei seg?”
2. “Jeg vet om et barn som virkelig, virkelig liker å være sammen med andre og være nær andre. Liker du å være sammen med andre og være nær andre?”
3. “Jeg vet om et barn som virkelig, virkelig liker å klemme andre. Dette barnet gir alltid klemmer til andre barn. Liker du å klemme andre?”
4. “Jeg vet om et barn som alltid trøster andre hvis de er triste eller lei seg. Pleier du å trøste andre som er lei seg?”

**Children’s Measures: Study 2**

*Communal Occupational Aspirations*

Instruction text:

English: “Now we would like to ask you about how much you would like to work in different jobs. You have probably thought about what you want to be when you grow up, and maybe you have decided what job you want to work in later. Either way, we would like you to try to picture how much you would like some different jobs now. Pick the answer that suits you best. How much would you like to be a [job] when you grow up?”

Norwegian: “Nå vil vi spørre deg om hvor mye du har lyst til å ha noen forskjellige jobber. Du har sikkert tenkt på hva du har lust til å bli når du blir stor, og kanskje du har bestemt deg for hvilken jobb du vil ha. Vi vil allikevel at du skal prøve å se for deg hvor mye du ville liket å ha noen forskjellige jobber nå. Velg det svaret som passer best for deg.”

Table 4
*Three Items for Measuring Children’s Communal Occupational Aspirations*

| Language | Item | Scale points | | | | | | | |
| --- | --- | --- | --- | --- | --- | --- | --- | --- | --- |
|  | Scale visualizations | 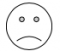 | 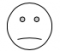 | | 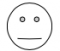 | | 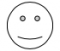 | | 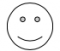 |
| English | How much would you like to be a nurse when you grow up? | Not at all | A little | | Some | | Quite much | | Very much |
|  | How much would you like to be a kindergarten teacher when you grow up? | Not at all | A little | | Some | | | Quite much | Very much |
|  | How much would you like to be a stay-at-home parent when you grow up? | Not at all | A little | | Some | | | Quite much | Very much |
| Norwegian | Hvor mye har du lyst til å bli en sykepleier når du blir stor? | Ikke i det hele tatt | | Litt | | Noe | Ganske | | Veldig mye |
|  | Hvor mye har du lyst til å bli en barnehagelærer når du blir stor? | Ikke i det hele tatt | | Litt | | Noe | Ganske | | Veldig mye |
|  | Hvor mye har du lyst til å være hjemme med barna dine når du blir stor? | Ikke i det hele tatt | | Litt | | Noe | Ganske | | Veldig mye |

*Communal Orientation*

Instruction text:

English: “Now we would like to ask you about what you like to do. Many people like to do different things, so there are no correct or wrong answers. No one from your school or your friends will now what you have answered”.

Norwegian: “Nå vil vi spørre deg om hva du liker å gjøre. Mange liker å gjøre forskjellige ting, så det er ingen riktige eller gale svar. Ingen fra skolen din eller vennene dine vil få vite hva du har svart”.

Table 5

*Three Items Measuring Children’s Communal Orientation*

| Language | Item | Scale Point | | | | |
| --- | --- | --- | --- | --- | --- | --- |
|  | Scale visualizations | 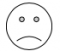 | 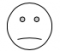 | 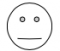 | 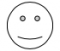 | 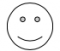 |
| English | Do you like to help other children when they are in pain? | Not at all | A little | Some | Quite much | Very much |
|  | Do you like to be with other children? | Not at all | A little | Some | Quite much | Very much |
|  | Do you like to comfort other children when they are sad? | Not at all | A little | Some | Quite much | Very much |
| Norwegian | Liker du å hjelpe andre barn når de har det vondt? | Ikke i det hele tatt | Litt | Noe | Ganske | Veldig mye |
|  | Liker du å være sammen med andre barn? | Ikke i det hele tatt | Litt | Noe | Ganske | Veldig mye |
|  | Liker du å trøste andre barn når de er lei seg? | Ikke i det hele tatt | Litt | Noe | Ganske | Veldig mye |

1. **Complete Regression Models**

Study 1

Complete model for testing moderation of age and gender on mothers’ occupation

Table 6

*Testing for moderation of children’s age and gender. Mothers’ occupation as predictor and children’s communal occupational aspirations as outcome.*

|  | B | SE(*B*) | *β* | t | *p* | *R^2^* |
| --- | --- | --- | --- | --- | --- | --- |
| Child gender | .13 | .21 | .10 | .59 | .559 | .11 |
| Child age | -.02 | .02 | -.12 | -.70 | .490 |  |
| Mothers’ occupation | -.93 | 2.36 | -.71 | -.39 | .697 |  |
| Child bilingualism | .02 | .10 | .02 | .16 | .871 |  |
| Income | .01 | .10 | .01 | .07 | .948 |  |
| Child age X mothers’ occupation | .02 | .04 | .78 | .42 | .676 |  |
| Child gender X mothers’ occupation | .18 | .34 | .22 | .52 | .605 |  |

Study 2

Complete model for testing moderation of age and gender of mothers’ occupation.

Table 7

Testing for moderation of children’s age and gender. Mothers’ occupations as predictor and children’s communal occupational aspirations as outcome.

|  | B | SE(*B*) | *β* | t | *p* | *R^2^* |
| --- | --- | --- | --- | --- | --- | --- |
| Child gender | .46 | .37 | .25 | 1.25 | .217 | .09 |
| Child age | -.00 | .01 | -.05 | -.20 | .846 |  |
| Mothers’ occupation | .65 | 1.38 | .32 | .47 | .638 |  |
| Child bilingualism | -.02 | .11 | -.02 | -.19 | .854 |  |
| Income | -.07 | .10 | -.08 | -.76 | .449 |  |
| Child age X mothers’ occupation | -.01 | .01 | -.30 | -.46 | .649 |  |
| Child gender X mothers’ occupation | -.06 | .44 | -.05 | -.13 | .898 |  |
| Dataset | -.11 | .25 | -.05 | -.44 | .658 |  |

**References**

Beere, C. A., King, D. W., Beere, D. B., & King, L. A. (1984). The sex-role egalitarianism scale: a measure of attitudes toward equality between the sexes. Sex Roles: A Journal of Research, 10, 563–576. [https://doi.org/10.1007/BF00287265](https://psycnet.apa.org/doi/10.1007/BF00287265)

Gaunt, R. (2006). Biological essentialism, gender ideologies, and role attitudes: what determines parents’ involvement in child care. *Sex Roles: A Journal of Research*, *55*, 523-533. <https://doi.org/10.1007/s11199-006-9105-0>

Olsson, M. (2021). *Causes and consequences of gender roles.* [Doctoral thesis, The Arctic University of Norway]. UiT Munin Open Research Archive. <https://munin.uit.no/handle/10037/20841>
